# Is a Government-Led Approach to Surveil Unhealthy Commodity Industries Feasible? Comment on "National Public Health Surveillance of Corporations in Key Unhealthy Commodity Industries – A Scoping Review and Framework Synthesis"

**DOI:** 10.34172/ijhpm.8601

**Published:** 2024-09-15

**Authors:** Angela Carriedo, Margarita Otero-Alvarez, Carmen Levis

**Affiliations:** ^1^World Public Health Nutrition Association, London, UK.; ^2^University of Nevada, Reno, NV, USA.; ^3^NCD Alliance, Washington, DC, USA.

**Keywords:** Public Health Surveillance, Unhealthy Commodity Industries, Organized Groups

## Abstract

Bennett and colleagues’ paper aims to synthesize the existing frameworks to identify and monitor unhealthy commodity industry’s (UCI’s) influence on health "to create a template surveillance system to be used by national governments across industries." In this commentary, we argue that to achieve a robust government-led national surveillance system, some challenges should be considered, such as (*a*) addressing power asymmetries between government and UCIs involved in policy-making, (*b*) evaluating competing interests among government constituencies to achieve policy coherence around health issues, and (*c*) contemplate whether governments rely on private or corporate donors and partners that may threaten financing and operationalization of the surveillance. Suggestions on how to overcome these challenges are beyond the scope of this commentary, but we discuss some cases of bottom-up approaches from organized groups aiming to hold UCIs accountable. We consider them to be emerging effective ways to support government-led initiatives and counter the long-lasting corporate power and negative impacts on public health.

## Introduction

 The need to synthesize, understand, and collide literature around the practices of commercial actors, particularly around unhealthy commodity industries (UCIs), has been suggested as a key way to move forward in research and action to counter UCI impacts on population and environmental health.^1-4^ Bennett and colleagues’ paper is a good synthesis of the frameworks that have emerged in recent years, and the categorization of common corporate actions (and inaction) to better understand and explore them.^5^

 The authors highlighted the similarities among health-impacting corporate practices of three industries: tobacco, alcohol, and ultra-processed food. They acknowledge that there are many other industries beyond the ones they focused on (eg, pharmaceuticals, firearms, and social media) that also contribute to commercial determinants of health (CDoH), and that there could be industry-specific practices they missed by their focus on said three industries.^5^ They also acknowledged that a full perspective of the public health harms of commercial actors requires going beyond commodities and low- and middle-income countries where governance practices may not be so well established.^6^ Furthermore, they acknowledge the need for increased research on this area from these regions, which is consistent with recommendations from the broader literature.^7^

 We argue that implementing governmental surveillance often presents a few challenges that need careful consideration related to (*a*) power asymmetries from UCI involvement in the public health policy process, (*b*) competing interests among government constituencies within each country impacting policy coherence around health issues, and (*c*) the reliance of public institutions on the private sector or corporate donors, which may threaten the surveillance’s financing, its operationalization and its use of adequate indicators. How to overcome these challenges is beyond the scope of this commentary, but we discuss some cases of effective bottom-up approaches from organized groups aiming for UCI accountability. These approaches have gained traction in advocating for corporate and government accountability, especially in regulating corporations, thereby challenging entrenched corporate influence and power in public policies.

 We consider, then, that people’s power, in the form of organized groups, is increasingly countering corporate power over public health governance. This avenue might be more conducive to supporting transparency in a governmental surveillance of UCI, which, by itself might conflict with governmental, commercial and economic interests.

## Potential Challenges of a Cross-commodity Framework for Surveillance

###  On Power Asymmetry and Structural Corporate Power

 In the current neoliberal paradigm, one potential challenge governmental surveillance might face when implemented is the power asymmetry between the government and UCI, especially if the UCIs are involved in the policy process. In Mexico, when the committee to evaluate the soda tax effectiveness was established by the Ministry of Health, several participating actors had conflicting interests and ultimately delayed the overall process and the setting of indicators.^8^ While the authors acknowledged that a full perspective of the public health harms of commercial actors requires going beyond the mentioned commodities and that we must consider practices and use of power,^6^ we argue that we must look even further into structural issues, norms, and current corporate policies that include close ties and agreements among UCIs, governments and public institutions.

 The institutionalized relationship between industry and government in tobacco-producing countries exemplifies this need. By leveraging the government’s norms regarding the tobacco industry as a key contributor to the economy, this UCI can position itself as a legitimate partner to the public sector. This has resulted in the industry being formally integrated into the policy processes via tobacco governance boards and committees, collaborations with agriculture ministries, research groups, and more.^9^ As Maani et al point out, corporate power has not been a mainstream focus of the public health community, and corporate actors’ role in influencing population health has likely been understated.^10^ However, as Wood et al mention, corporate power “over” the public health agenda has not been a key factor in pushing back among public health actors.^11^

 Considering what Wood et al argue on corporate power, which is based on Foucault’s forms of power: its origins, nature, and manifestations,^12^ this needs better scrutinizing, and using the frameworks synthesized within Bennet and colleagues’ paper can help detect, organize, and strategize against it. Nevertheless, powerful forces that overlook health-related issues, like trade policies, international investment agreements, and neoliberal economies or structural power “over” governments, can be challenging to surveil. This exacerbates the challenges of dismantling these powerful forces, with structural (Fuch’s framework) power being far more complicated than instrumental and discursive power.^13,14^

 These issues have largely been overlooked and under addressed among scholarly work and public practice, particularly from the health perspective. However, Lee et al attempt to measure the influence of such powerful forces on the health and well-being of populations in their proposed framework to measure CDoH and disease.^2^ They include indicators that they grouped into market strategies, non-market strategies, political and economic systems, stratification, governance, and norms. Each of these domains includes examples that resemble those found within the frameworks reviewed by the authors. Lee and colleagues’ framework, in particular, tries to quantify corporate actions and “structural influences” on health.^2^ We recognize that what we are highlighting has been previously acknowledged, but we strongly encourage the public health community and activists to continue highlighting how entrenched the current status quo of corporate power is and the time it would take to shift paradigms in situations where independent institutions and governance structures exercise power over the “public health” agenda.

###  Increased Reliance on Private Institutions

 Another challenge to consider with a government-led surveillance system is governments’ increased reliance on private institutions. While the authors provide strong examples of independent systems such as the: “Revolving Door Watch,” “Impact Assessment Expert Group,” and “Global Tobacco Interference Index,” they are not completely government-led initiatives. They include the involvement of different actors, many public-led organizations, consumer groups, and non-governmental organizations. Thus, we suggest that, in addition to a government-led system, surveillance should also stem from organizations leading bottom-up approaches. This can strengthen surveillance efforts by holding governments accountable, amplifying the public’s voice, as well as exercising the right to information, the right to health, and the protection of the state.

 Government-led systems must overcome structural changes to implement, maintain and evaluate effective surveillance systems free from conflict of interest and industry interference. Bottom-up approaches, while facing challenges such as financial independence, also tend to have fewer problems associated with institutional structures such as those faced by public institutions, particularly in low- and middle-income countries economies.^14,15^

###  Lack of Policy Coherence Among Government Sectors

 While Bennet et al proposed a cross-sectoral governmental approach to UCI corporate activities, including health, agriculture, finance, trade, and taxation, we acknowledge the lack of policy coherence and coordination across government sectors could be a potential challenge. For example, it is not common that ministries align to prioritize public health outcomes to the same extent. Competing interests exist among, for instance, the Ministries of Finance, Commerce, and Agriculture, as well as the different legislators who influence policies, such as approving a UCI surveillance system. Sometimes, public health outcomes align with financial outcomes, such as sugar-sweetened beverages taxes, which have double-duty action. Sugar-sweetened beverages taxes not only improve population health but also generate revenue and potentially reduce long-term associated healthcare costs and productivity losses.^16^ However, other policies related to the surveillance of corporations may not be a priority outside the Ministry of Health. For example, food warning labels may be backed by the Ministry of Health but could be perceived as a threat to the economy by some legislators, potentially due to their ties to corporations or other interests, as was the case in Argentina, which delayed its implementation.^17^

 Additionally, many local governments have close ties to UCIs, some of which take the form of public-private partnerships and other forms of collaboration.^18^ These ties and interests can become a politician’s conflict and often result in them pursuing policies leading to an economic, social or commercial outcome that promotes their position or personal interest rather than pursuing policies that promote and protect health, such as a surveillance system of UCIs looking at policies, products and actions that threaten health outcomes.

 Nevertheless, some examples of cross-ministerial collaboration have shown to be successful. Positive cases often involve a policy entrepreneur, usually with personal motivations to push for a public health agenda and a vision for double-duty actions.^19^ The 2021’s United Nations Food Systems Summit resulted in designing some policy pathways for sustainable food systems where interministerial government officials were involved from each country.^20^ That scheme has been challenged in some African countries but has been successful in others, such as Brazil, particularly in delivering school meals.^21^ It is yet to be seen if it is successful in other countries. Also, the Framework Convention on Tobacco Control is a solid example of muti-ministerial collaborations in many countries, but it is yet to be seen if a surveillance mechanism could work, particularly if recommended by the United Nations or backed by a binding treaty.

###  Bottom-up Approaches as a Reference of “Surveillance” on Corporate Actions

 The authors mentioned that monitoring the health impacts of UCIs has mainly been a task of civil society and academia. We acknowledge and praise the emerging bottom-up approaches of organized groups advocating for corporate and government accountability, reflecting a unified effort to dismantle powerful UCIs. Some of these initiatives also aim to regulate corporations and challenge their influence, countering structures prioritizing profit over public well-being. We believe that the power of the people can challenge entrenched corporate governance that neglects public health. This approach can enhance governmental health surveillance, which could be swayed by governmental commercial and economic interests.

 Here we provide two examples of successful cases that display this effort and have equally worked against different UCIs, suggesting that with similar strategies, all corporations can be targeted.

 Recently in the United Kingdom, a WhatsApp group created by local parents seeking to protect their children from the dangers and distractions of smartphones turned into a national movement across Britain, with a regional WhatsApp group now in every country in the United Kingdom.^22^ Their campaign, Smartphone Free Childhood, plans to present their cause to Parliament and lobby for a ban on smartphones among young children.^22^ While legislation on this issue has not yet materialized, the fact that discussions on this topic are underway is a commendable step in the right direction.

 On the other hand, Colombia has strong consumer advocacy and legal groups focused on the constitutional rights of children and supporting healthy eating.^23^ Policy wins, including the implementation of octagonal front-of-package warning labels and two fiscal policies that cover taxes on ultra-processed sugary beverages and ultra-processed food products, were made possible due to years of aggressive advocacy efforts by local partners such as Red Papaz, Dejusticia, Colectivo de Abogados “José Aguilar Restrepo” (CAJAR), and Food First Information and Action Network (FIAN) Colombia, challenging periods of industry interference throughout the policy-making process.^24^ The case was also supported by several academic and civil society organizations from Latin America and the Caribbean that submitted amicus briefs to the court, asserting the constitutionality of the law and underscoring the significance of the decision for the region.^24^

## Discussion

 The authors’ proposal for a cross-cutting framework supporting national government monitoring of corporations’ UCI’s impact on health is significant and a novel strategy to keep corporations accountable. Nevertheless, a government-led surveillance strategy would face some potential challenges. Its success relies on several assumptions that may need to be considered. Firstly, not all government institutions prioritize public health in policy-making. Secondly, this approach to UCI scrutiny may not suffice without addressing other interests and powerful political economy issues of other involved constituencies. Thirdly, governmental autonomy at the sub-national level (crucial for decision-making regarding UCI partnerships) might be limited. Finally, government-led investigation and monitoring require adequate resources, yet the public sector increasingly relies on private financing. Research shows that public and intergovernmental institutions are relying more often on private financing, including philanthropy, and mainly from commodities that are either UCI or have very close ties to them, such as pharma (to food and tobacco), technology (to food, tobacco, alcohol, and fossil fuels), or agro-industry (to tobacco and food).^25,26^ This simplifies corporate power over public health-focused institutions.

 Additionally, as the authors successfully point out, a wide range of institutions currently monitor the impact of UCI corporate practices (political, promotional, social/community), however, “these efforts are disparate, with no identified initiatives monitoring a complete series of practices over multiple UCIs, despite the need of a cross-industry approach identified in the literature.”^5^ We question how feasible it is to have a cross-industry approach considering the challenges of the current institutions and interests.

 Despite different frameworks identifying similar corporate practices, targeting different commodities and industries to monitor and survey might be challenging. Policy-makers’ and constituencies’ priorities and goals vary, posing challenges to uniform monitoring efforts across commodities. Academics and advocates look for policy coherence and opportunities to integrate health in all policies, but we rarely see this in current practice. The next step will be to operationalize such concepts and frameworks in a practical way that middle-range bureaucrats can implement. Alternatively, civil society organizations could continue their effective monitoring role despite not following a particular framework.

 Kelly et al propose a framework to measure CDoH, with some political and economic indicators that can potentially measure UCI’s influence on the economy, policies, and environment.^2^ It would be ideal to evaluate its implementation and validation at national or subnational levels to further its application.

 In conclusion, implementing governmental surveillance poses challenges due to the power asymmetries between government and UCIs in the current neoliberal paradigm, increased reliance of governments on private institutions, policy incoherence, competing government interests, and potential issues with surveillance’s financing and operationalization.^13,14^ We, therefore, view the issue the authors raise as the exertion of power ‘over’ political and societal health interests, deeply intertwined with the actions and practices that are part of CDoH. While well recognized, these entrenched dynamics are not changed in a day, but over years, and with strong emerging bottom-up approaches, these changes of power and cries for transparency and corporate accountability are leveraging the scales, so it is worth supporting such groups and building coalitions to support and protect them.

## Ethical issues

 Not applicable.

## Conflicts of interest

 Authors declares that they have no conflicts of interest.
